# Supplementary material for: Genome assembly with in vitro proximity ligation data and whole-genome triplication in lettuce
Source: Nat Commun. 2017 Apr 12;8:14953. doi: 10.1038/ncomms14953 (PMC5394340; doi:10.1038/ncomms14953)
Supplement: Supplementary Information — Supplementary Figures, Supplementary Tables, Supplementary Note and Supplementary References [file ncomms14953-s1.pdf]

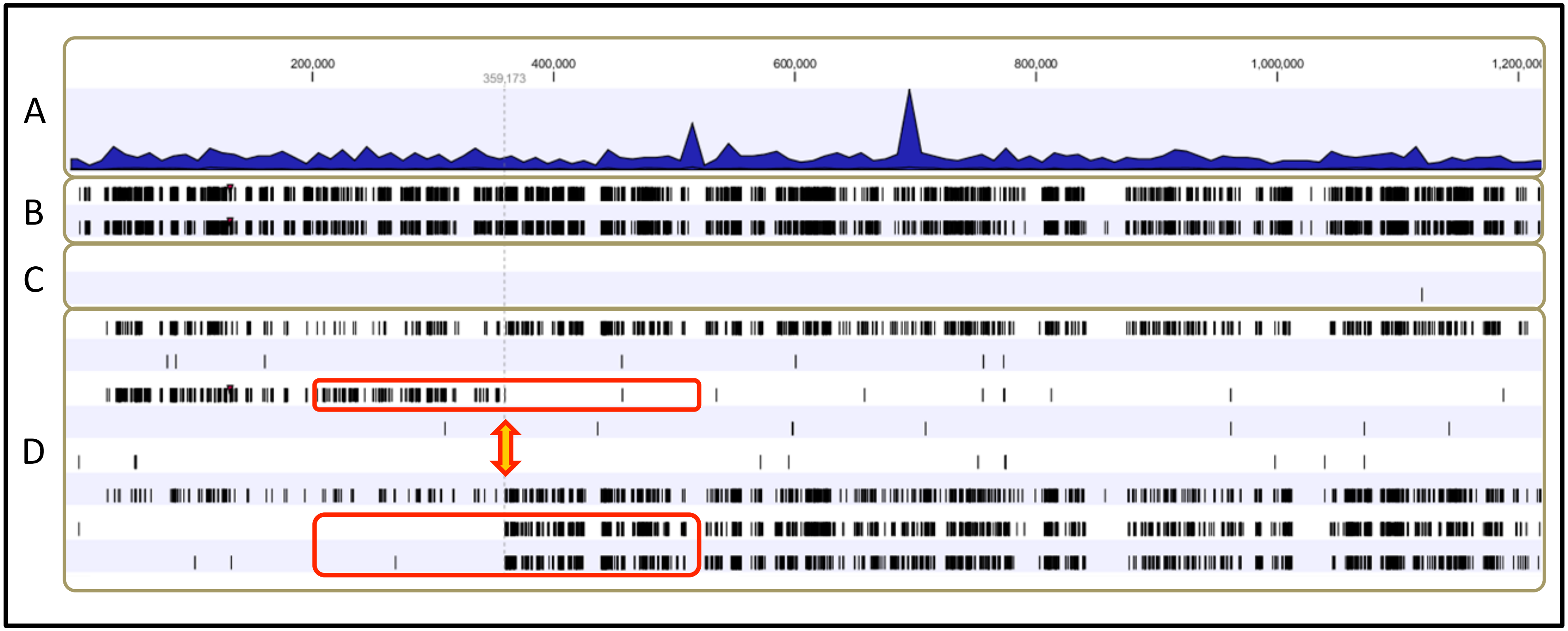

Supplementary Figure 1. **Example of identification of chimeric scaffolds**. Visualization of Illumina read alignment and variant calls with CLC Genomics Workbench for the controls and a set of RILs to a region of chimeric scaffold. Track A displays average genomic read coverage. Panels B, C and D display SNP density across scaffold sequence. Panel B: two control samples of *L.serriola* genotype; panel C: two control samples of *L.sativa* genotype; panel D: set of eight RILs. Red box indicates scaffold region with frequent transition of SNP density from *L.sativa* to *L.serriola* genotype within a set of RILs.

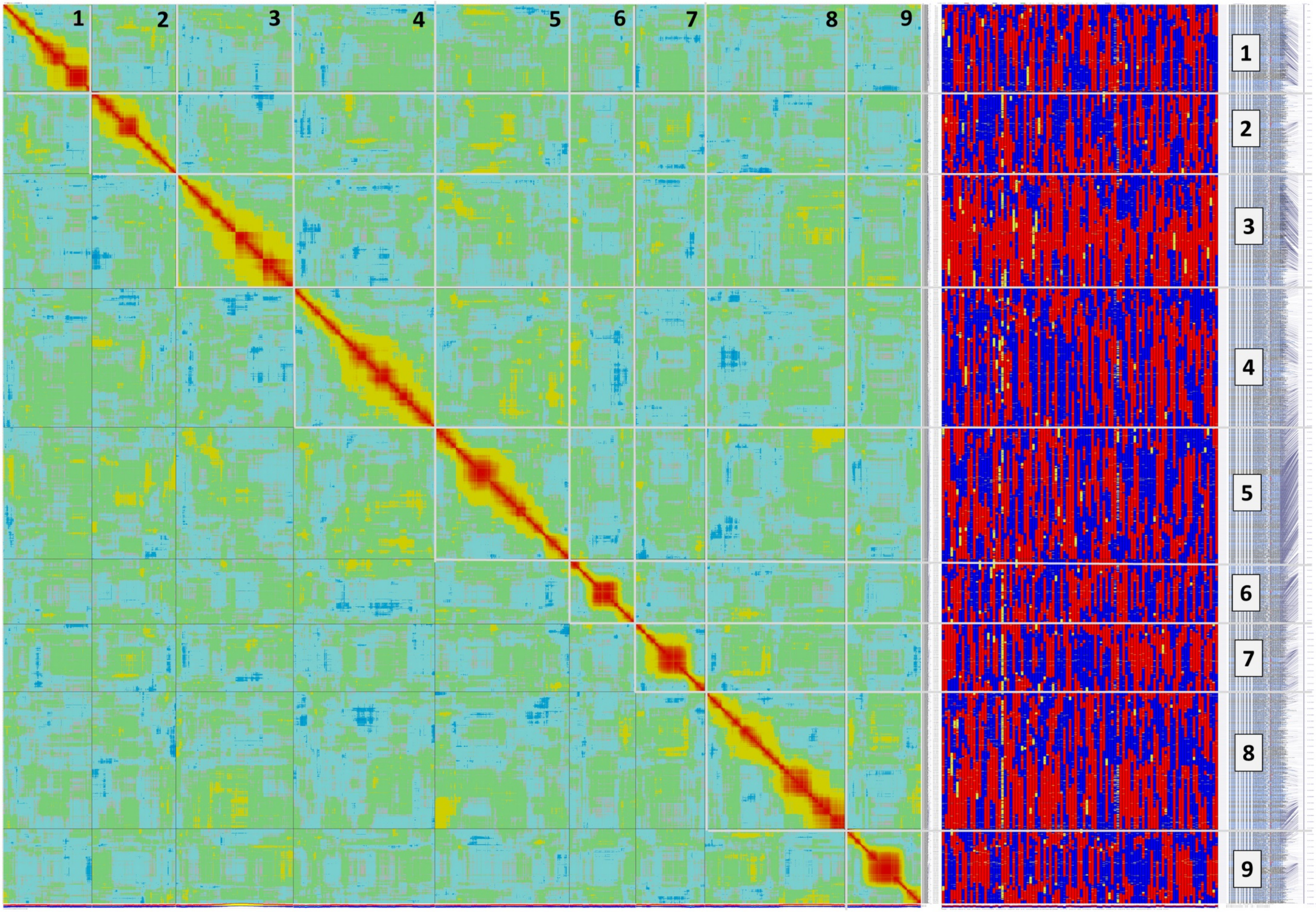

Supplementary Figure 2. **Genetic map for 9,140 SOAPdenovo scaffolds for entire *L. sativa* cv Salinas genome**. Left: Linkage heat plots of concatenated 9 chromosomes. Right: Graphical genotyping across the 9 chromosomes.

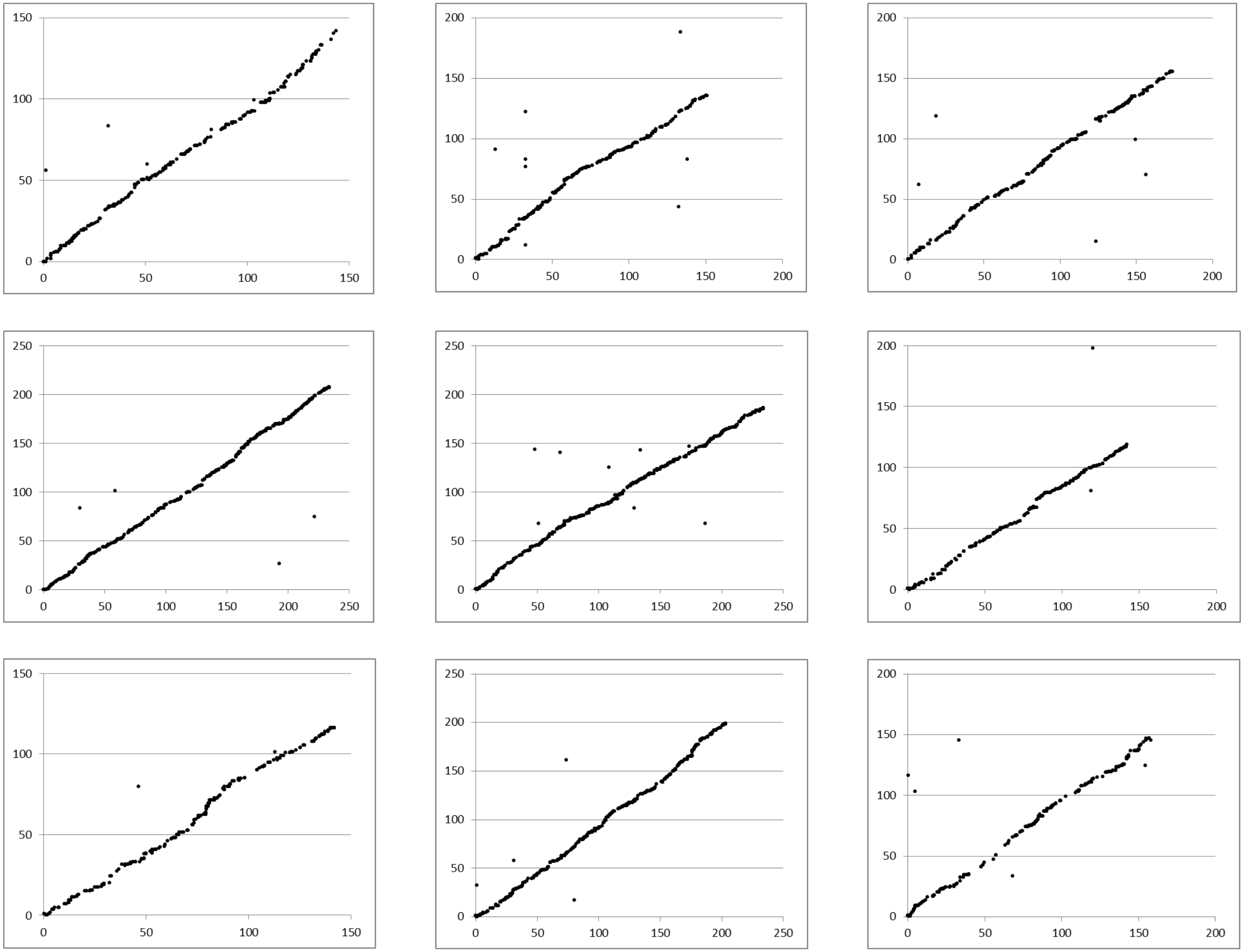

Supplementary Figure 3. **Collinearity between the *L. sativa* cv Salinas GBSS genetic map and the Affymetric based map**

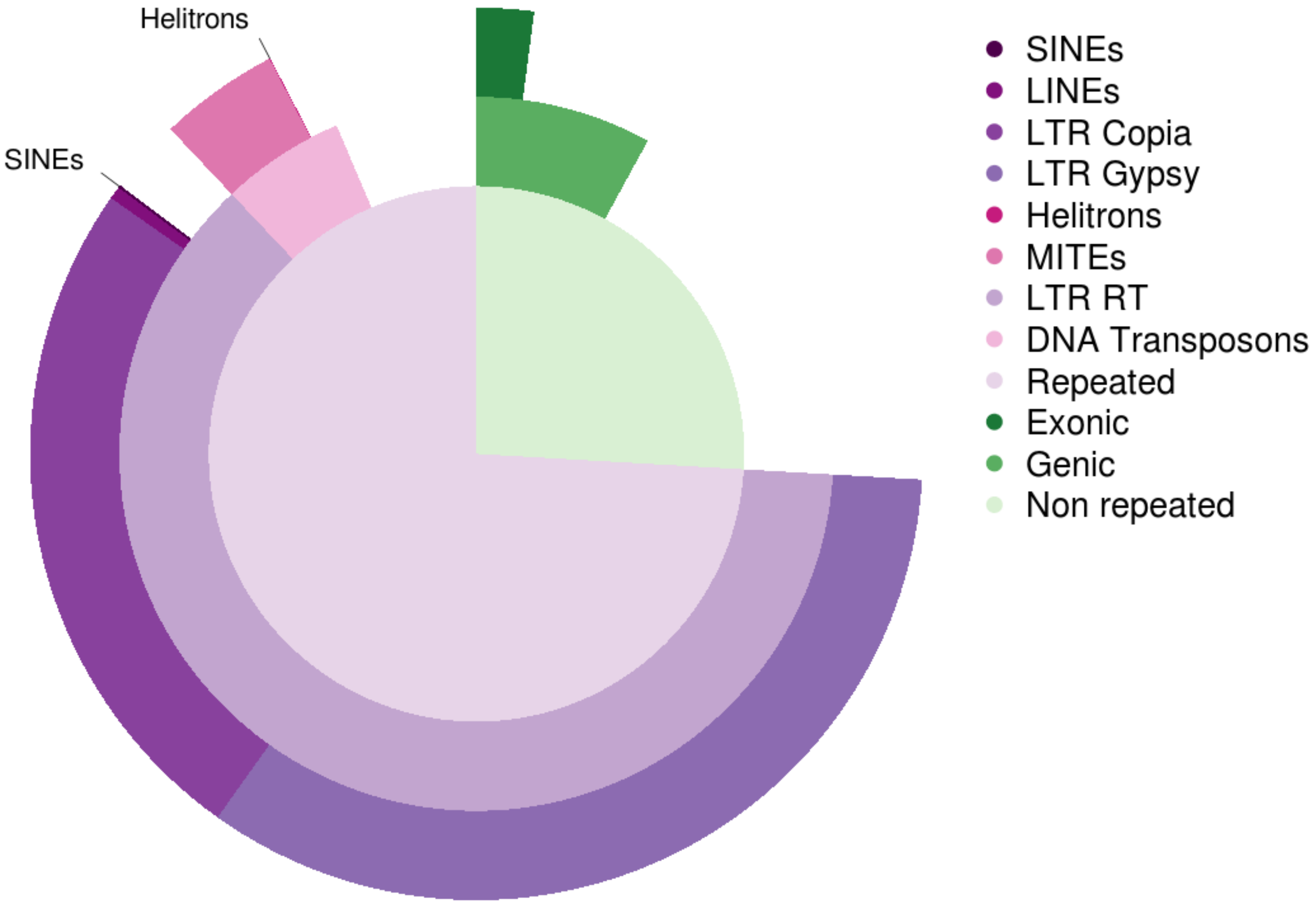

Supplementary Figure 4. **Composition of the *L. sativa* cv Salinas genome based in feature type**

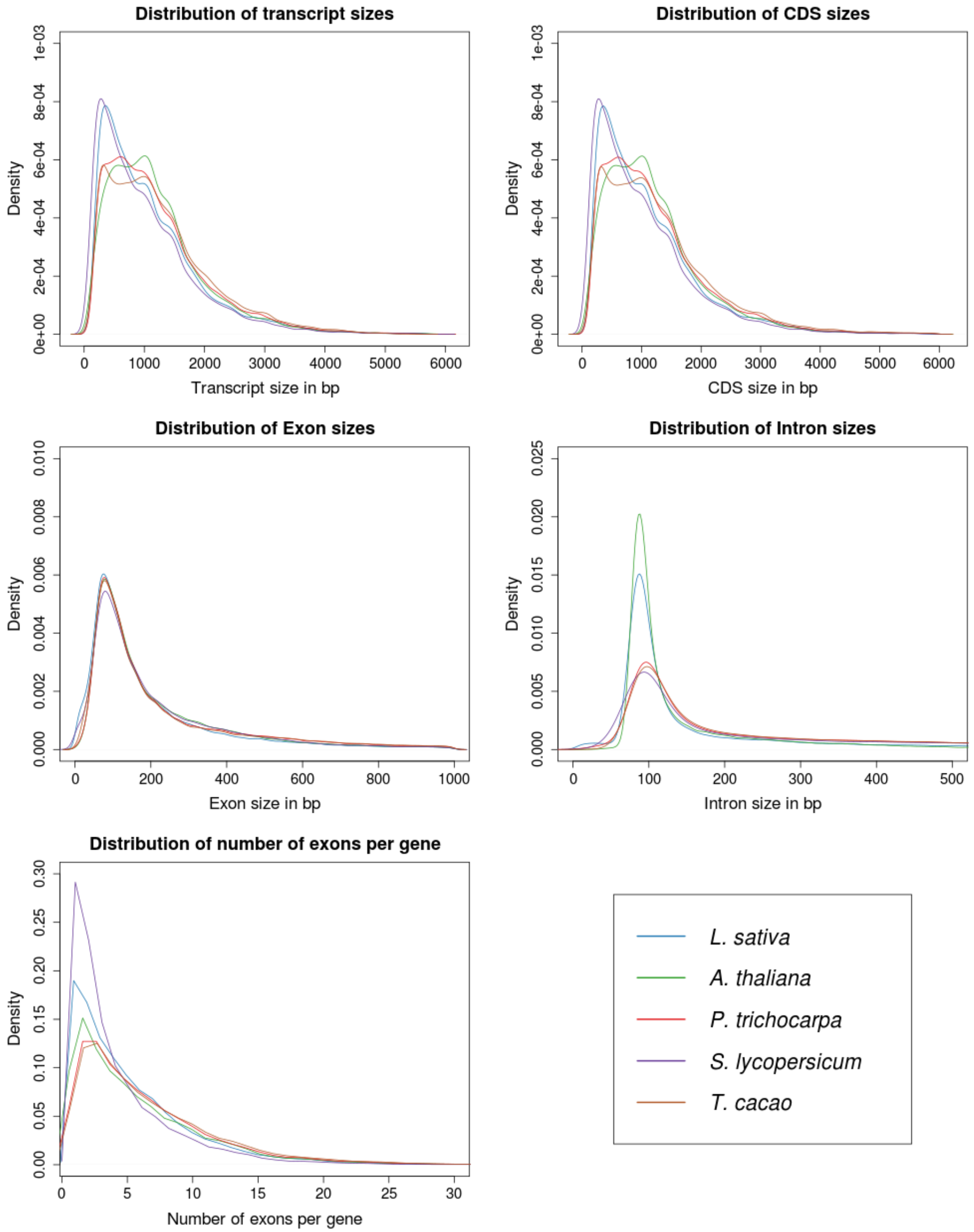

Supplementary Figure 5. **Comparison of the distribution of different genomic features of *L. sativa* against other published genomes**. All measurement were calculated based in annotation from Phytozome V9 with in-house scripts. Although there are large differences in genome size across these species no significant differences are visible in the distribution of gene-related (exons/introns) features.

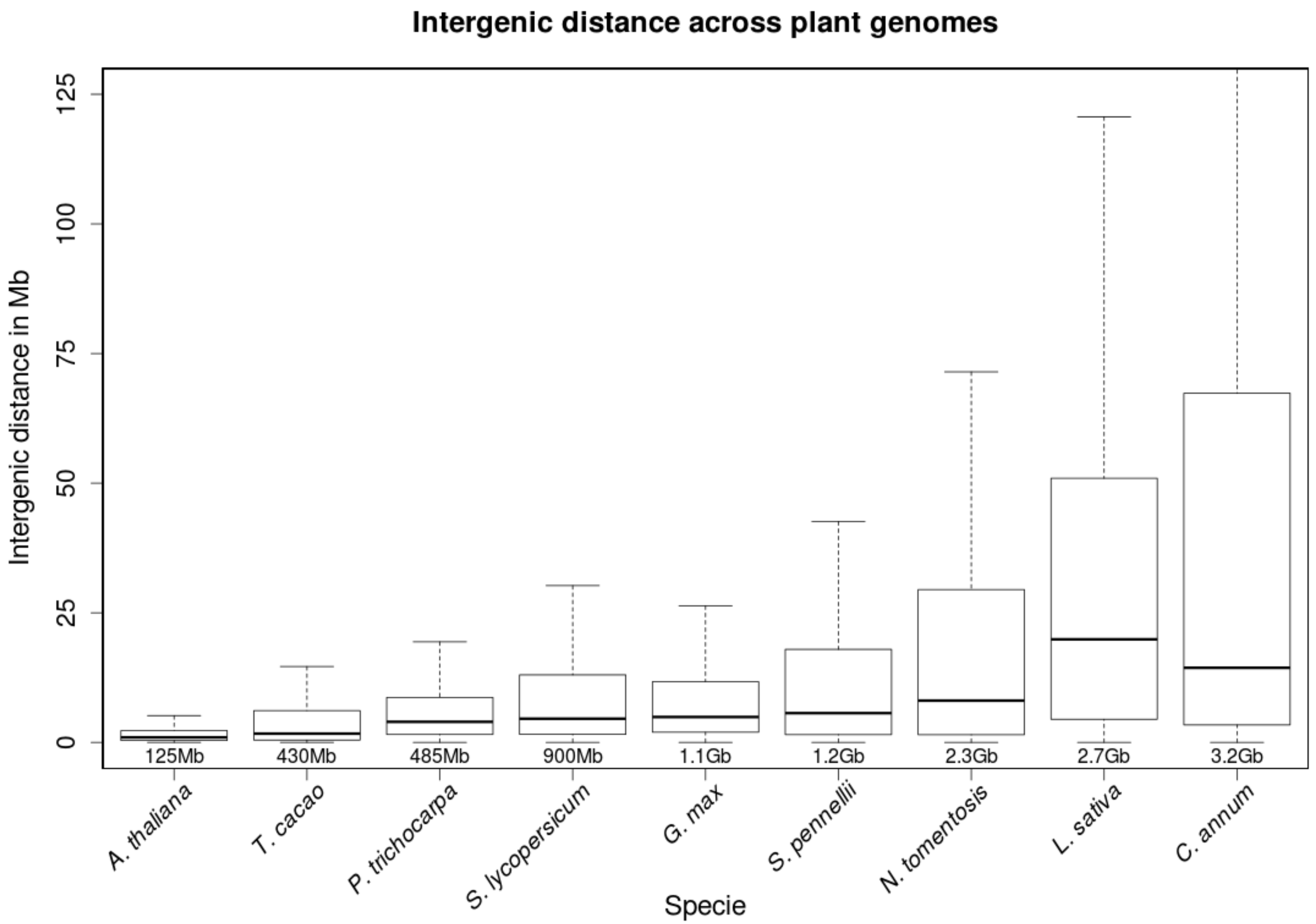

Supplementary Figure 6. **Boxplot of intergenic distance across published plant genomes and lettuce**. Number below the box shows estimated genome size. Intergenic distances were calculated based in annotation from Phytozome V9 with in-house scripts.

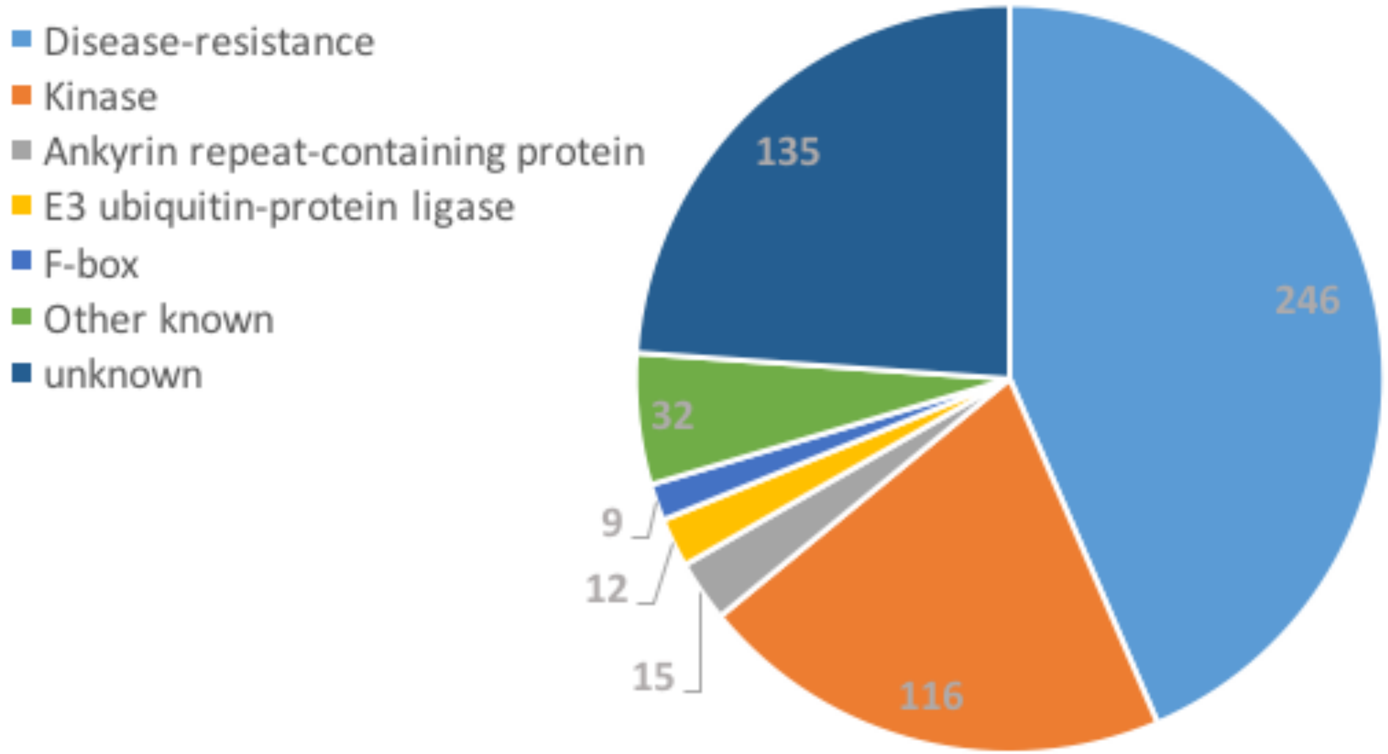

Supplementary Figure 7. **Loci in lettuce producing phased, secondary siRNAs (phasiRNAs)**. A total of 565 phased siRNA-generating loci (*PHAS* loci) were identified in lettuce using the set of ten small RNA libraries. A total of 565 *PHAS* loci were identified, the majority of which (430 loci) were annotated as protein-coding genes. *NB-LRR* disease resistant genes were the largest class of genes generating phasiRNAs (246); the second largest category was kinases, from 116 genes. A large group of 135 loci were not classified due to missing annotations; this may include orthologs of known tasiRNAs (*TAS* loci).

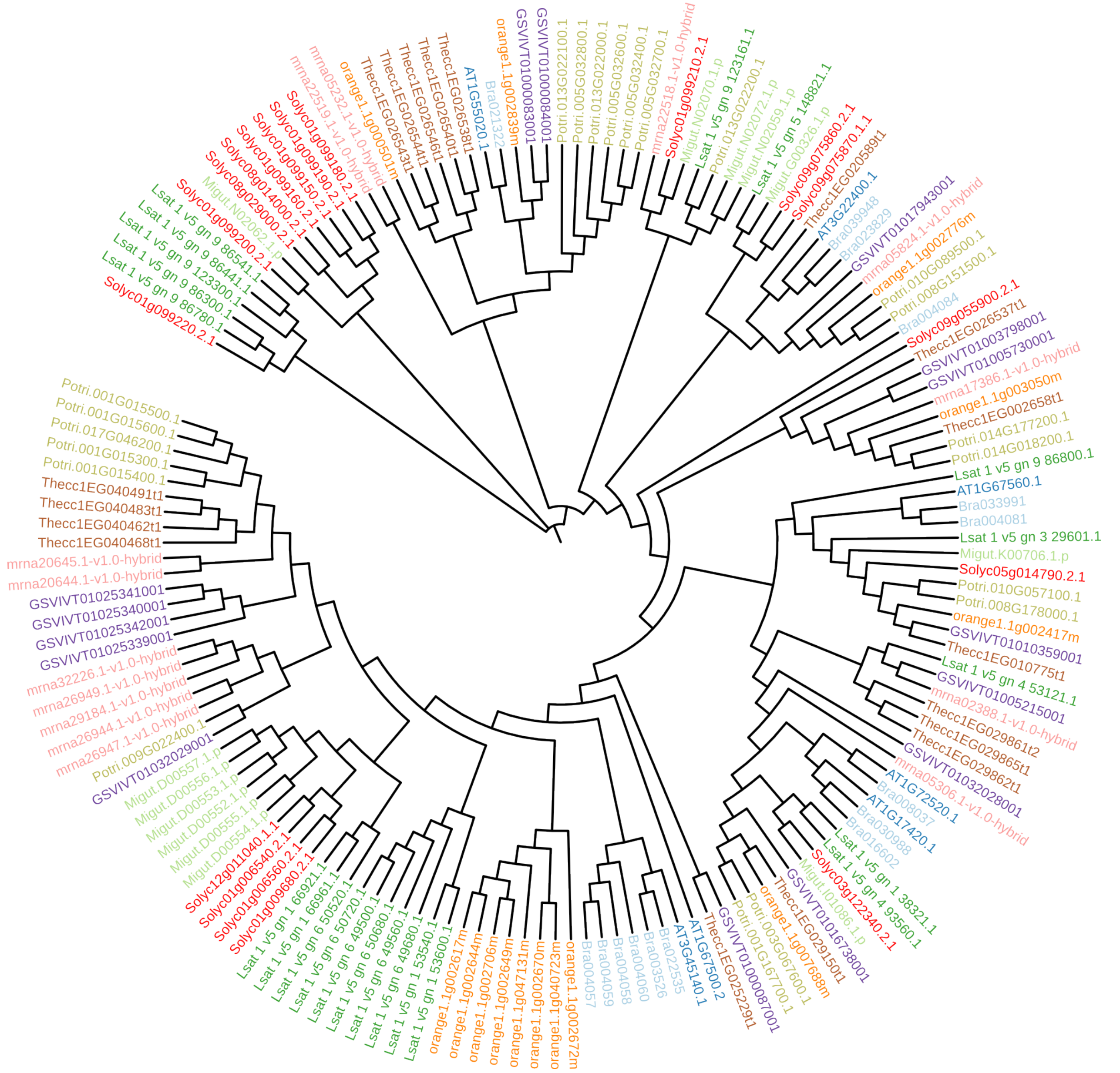

Supplementary Figure 8. **Phylogenetic tree of LOX-like genes across 10 plant genomes**. Color coding done by specie. *L. sativa*: Green, *S. lycopersicum*: Red, *M. guttatus*: Light green, *C. sinensis*: Orange, *B. rapa*: Light blue, *V. vinifera*: Purple, *A. thaliana*: Blue, *F. vesca*: Pink, *T. cacao*: Brown, *P. trichocarpa*: Olive

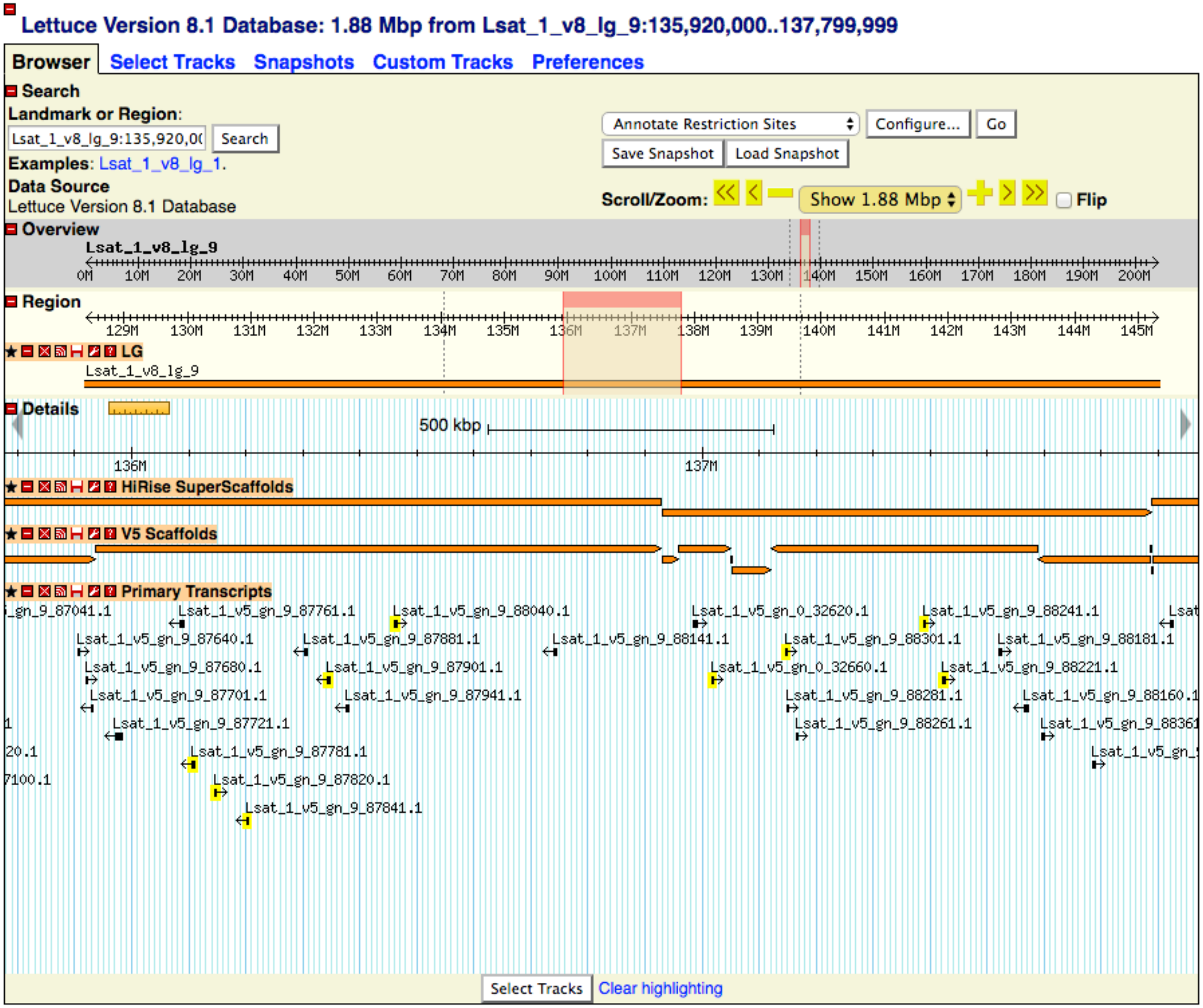

Supplementary Figure 9. **Structure of the REF gene cluster on *L. sativa* LG9**. REF genes are highlighted on yellow. Left side of the cluster was easily assembled on a single SOAPdenovo scaffold (V5 Scaffolds), while the right section required the Hi-Rise information to place the genes in a more accurate arrangement.

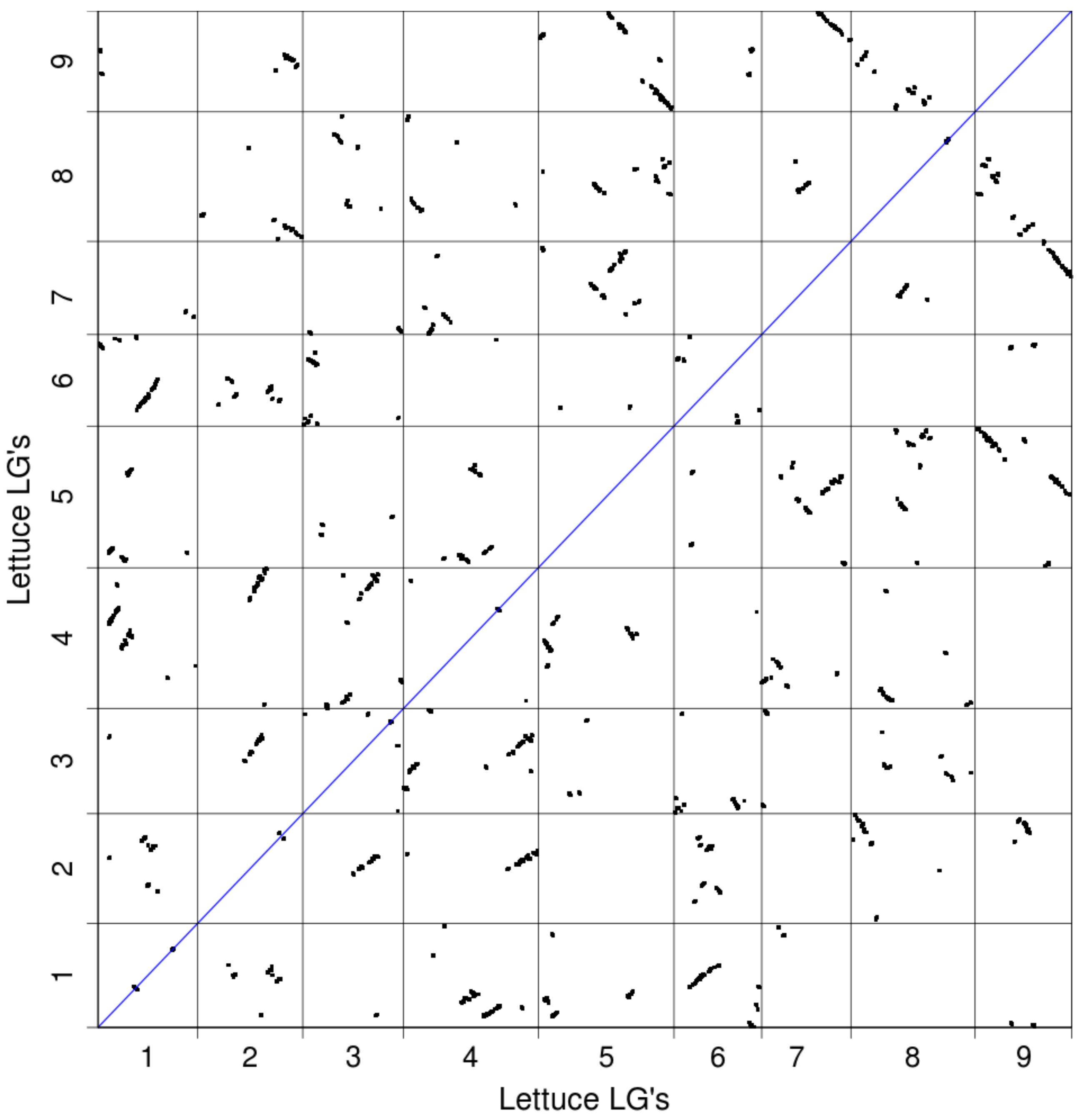

Supplementary Figure 10. **Intragenomic synteny of the *L. sativa* genome**. Diagonal blue line is the hit of each gene against itself. Black dots are syntenic hits between two different genes.

Supplementary Figure 11. **Gene tree of the *L. sativa* TCP transcription factor family**.
Green branches: *Cycloidea*-like genes
Green genes: Genes with conserved syntelog
Blue genes: Genes within triplicated region without conserved syntelog

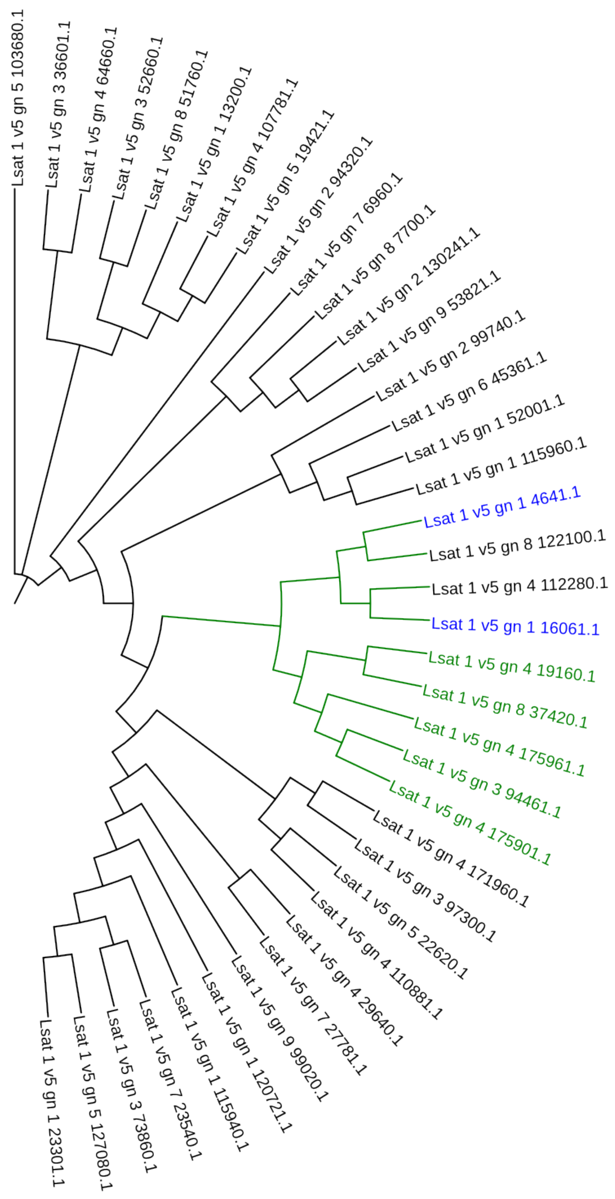

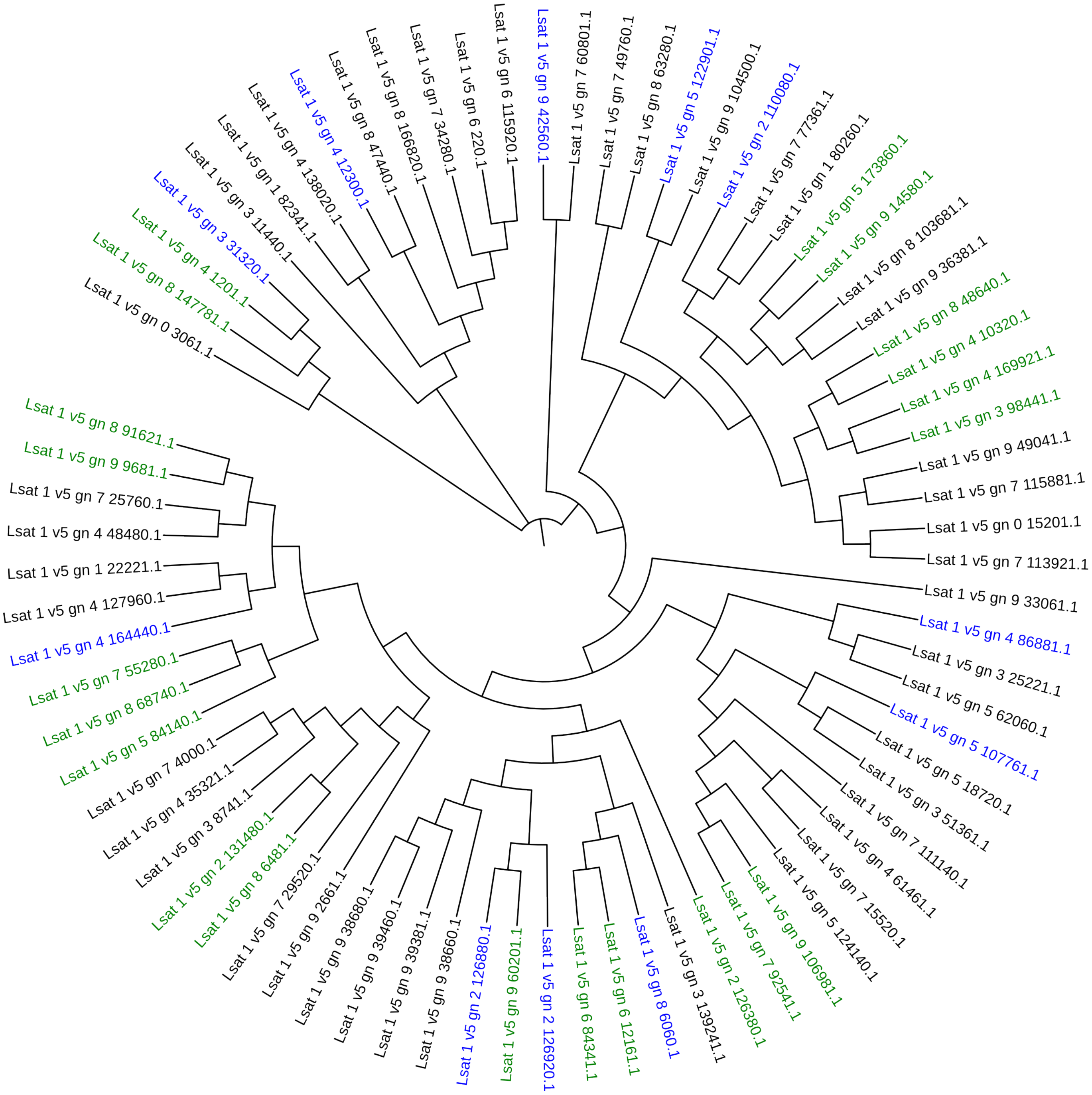

Supplementary Figure 12. **Gene tree of the *L. sativa* WRKY transcription factor family**. Green genes: Genes with conserved syntelog Blue genes: Genes within triplicated region without conserved syntelog

Suppplementary Table 1. **Illumina read library statistics**

Insert size	Read length	Raw Reads			Assembled Reads		
		Total data (Gb)	Sequence coverage (X)	Physical coverage (X)	Total data (Gb)	Sequence coverage (X)	Physical coverage (X)
170bp	100	68.20	25.26	21.47	61.50	22.78	19.36
500bp	100	55.6	20.59	51.48	46	17.04	42.59
800bp	100	52.4	19.41	77.63	39.5	14.63	58.52
2kb	49	38.7	14.33	292.52	23.9	8.85	180.65
5kb	49	27	10.00	510.20	15	5.56	283.45
10kb	49	37.6	13.93	1421.01	6.6	2.44	249.43
20kb	49	11.2	4.15	846.56	2.2	0.81	166.29
40kb	49	7.3	2.70	1103.55	1.1	0.41	166.29
Chicago1	100	62.7	23.22	NA	NA	NA	NA
Chicago2	100	71.44	26.46	NA	NA	NA	NA

Sequence Coverage	Total data / estimated genome size
Physical Coverage	((Total data/(2*read length)) * insertsize) / estimated genome size

Supplementary Table 2. **Statiscis for the *L. sativa* genome assemblies**

	SOAPdenovo				HiRise	
	Contig		Scaffold		Superscaffold	
	Length	Number	Length	Number	Length	Number
N50	11,737	57,382	476,083	1,445	1,769,135	385
N90	1,937	230,301	117,623	5,237	360,301	1,520
Longest	118,200	1	3,094,459	1	12,266,363	1
Total size	2,328,562,194	1,040,974	2,383,281,025	21,686	2,384,129,484	11,474
> 100 bp	2,328,182,901	1,035,304	2,383,281,025	21,686	2,384,129,484	11,474
> 1 Kb	2,169,937,518	281,955	2,383,281,025	21,686	2,384,129,484	11,474
> 2 Kb	2,090,021,108	227,413	2,372,527,449	13,704	2,376,078,455	5,448
> 10 Kb	1,313,624,757	71,166	2,353,592,287	9,415	2,367,598,603	3,325
> 100 Kb	436,902	4	2,186,283,040	5,617	2,330,126,513	2,391
> 1 Mb	NA	NA	417,375,212	303	1,658,540,163	741
GC content	38%	-	35%	-	35%	-
N content	0%	-	7%	-	7%	-
CEGMA score	97.18/97.58				97.58/97.58	
%UCOs	100%				100%	

Supplementary Table 3. **Statistics of the HiRise scaffolding iterations**

	HiRise 1 lane	HiRise 2 lanes
Chicago Library Coverage	30.3X	72.0X
Total Length (Mb)	2383.9	2384.3
Total Num Sequences	15,413	11,438
Total Num Sequence Decrease	27.2%	46.0%
N50 Scaffold (Num/Length)	1.05Mb / 650	1.8 Mb / 383
N50 Fold Increase	2.2	3.78
N90 Scaffold (Num/Length)	0.232Mb / 2,483	0.366Mb / 1,504
N90 Fold Increase	1.98	3.12
Mean Sequence Length (kb)	154.7	208.4
Num of sequences > 1Mb	689	746
Sequence in Scaffolds >1Mb	1.23GB	1.67Gb
Perc Sequence >1Mb	51.69%	70.11%

Supplementary Table 4. **Complete statistics for predicted geneset**

	Primary transcripts	All transcripts
Num gene models	38919	62670
Num monoexonic gene models	10131	11461
mean transcript length	1264.78	1328.3
median transcript length	1032	1095
StDev transcript length	1024.21	1040.73
min transcript length	147	147
max transcript length	16266	16276
mean CDS length	1050.76	1135.51
median CDS length	829	915
StDev CDS length	865.06	919.65
min CDS length	68	68
max CDS length	15877	16069
mean num exons	4.54	5.44
max numexons	76	77
total num exons	176560	340913
mean exon length	278.79	244.18
median exon length	146	131
StDev exon length	392.23	346.55
min exon length	0	0
max exon length	12526	12526
mean intron length	338.38	317.61
median intron length	106	107
StDev intron length	1041.01	894.4
min intron length	5	5
max intron length	49398	49398
Mean IGD	39506.95	
Median IGD	19881	

Supplementary Table 5. **Summary of OrthoMCL clustering of proteins from *L. sativa* and 9 published plant genomes**

Specie	Total number genes	Clustered genes	Ungrouped genes	Number families	Unique families	Genes in unique families	Mean number genes per family	Cegma score (complete - partial)
A. thaliana	27,416	23,348	4,068	16,411	397	1,179	1.42	96 - 100
B. rapa	40,905	31,987	8,918	16,652	843	2,829	1.92	96 - 99
C. sinensis	25,379	20,031	5,348	14,511	527	1,750	1.38	82 - 96
F. vesca	32,831	24,025	8,806	14,127	1,326	6,131	1.70	88 - 96
L. sativa	38,917	29,511	9,406	14,792	1,617	7,035	2.00	98 - 100
M. guttatus	28,140	23,382	4,758	14,239	631	2,255	1.64	83 - 91
P. trichocarpa	41,335	32,736	8,599	15,696	935	2,742	2.09	82 - 94
S. lycopersicum	34,727	25,036	9,691	14,720	953	4,147	1.70	74 - 93
T. cacao	29,452	22,890	6,562	15,377	542	2,535	1.49	88 - 96
V. vinifera	26,346	18,830	7,516	13,515	652	1,782	1.39	65 - 92

Supplementary Table 6. **Statistics for all the detected triplicated regions on the *L. sativa* genome**

	Subgenome	Total length	Total num genes	Total num conserved genes	Repeat content	Gene content
TripA		52,493,579	1,113	277	70.5%	6.1%
lg_1	I	11,150,080	351	81	66.3%	8.0%
lg_2	II	20,465,239	383	100	71.3%	5.3%
lg_6	III	20,878,260	379	96	75.2%	4.5%
TripB		131,709,655	2,049	476	73.1%	5.3%
lg_1	II	20,001,692	614	175	68.2%	8.7%
lg_4	III	62,408,438	779	143	75.1%	3.3%
lg_5	I	49,299,525	656	158	75.2%	4.7%
TripC		25,868,676	451	95	71.7%	4.7%
lg_1	I	5,150,029	137	31	68.9%	7.5%
lg_6	II	9,080,480	129	35	71.6%	3.4%
lg_9	III	11,638,167	185	29	74.7%	3.7%
TripD		98,641,135	1,399	310	75.8%	4.7%
lg_2	III	13,269,003	381	105	69.3%	7.9%
lg_3	II	39,999,969	450	85	78.7%	3.4%
lg_4	I	45,372,163	568	120	78.8%	3.1%
TripE		57,725,471	1,407	384	68.8%	6.5%
lg_2	II	17,076,282	568	155	65.0%	8.4%
lg_8	III	18,114,857	460	133	69.9%	6.1%
lg_9	I	22,534,332	379	96	72.8%	4.5%
TripF		51,618,194	972	212	72.4%	5.0%
lg_3	III	10,837,432	271	61	71.3%	5.7%
lg_4	I	17,330,244	351	77	72.6%	5.1%
lg_8	II	23,450,518	350	74	73.5%	4.2%
TripG		34,051,705	752	182	70.7%	6.1%
lg_3	II	15,411,526	367	76	71.3%	5.7%
lg_6	I/III	18,640,179	385	106	70.5%	6.3%
TripH		55,624,185	915	210	74.4%	4.6%
lg_5	II	22,572,762	311	71	75.7%	3.4%
lg_7	III	14,577,251	291	66	73.4%	5.7%
lg_8	I	18,474,172	313	73	74.6%	4.3%
TripI		79,232,276	1,429	207	74.9%	5.0%
lg_5	III	26,239,248	477	4	75.1%	5.0%
lg_7	II	28,372,473	523	115	73.7%	4.9%
lg_9	I	24,620,555	429	88	76.0%	5.2%
TripJ		64,346,043	1,645	459	68.3%	7.0%
lg_5	III	22,904,931	521	136	70.2%	5.5%
lg_8	II	22,959,146	498	151	71.8%	5.1%
lg_9	I	18,481,966	626	172	62.8%	10.5%
Grand total		651,310,919	11,816	2912	71.9%	5.6%

Supplementary Note 1

Detailed materials and methods

Library preparation and sequencing

DNA was extracted using a modified CTAB method[1] from seedlings of *L. sativa* cultivar (cv) Salinas grown in the dark under sterile conditions in Magenta boxes with Hoagland's nutrient solution. Seven genomic libraries of inbred, homozygous *L. sativa* cv. Salinas were constructed with insert sizes of 170, 500, and 800 bp, and 2, 5, 10, 20 and 40 Kb and sequenced using 100 + 100 paired-end (PE) reads on the Illumina HiSeq 2000 platform to generate 298 Gb of sequence. Filtering to remove low quality reads (>25 bp with under Q20), reads with adapter contamination (>10 bp adapter), read with ambiguous bases (>10% N's) and error correction provided 198.5 Gb of clean data for assembly.

The 99 recombinant inbred lines (RILs) derived from *L. sativa* cv. Salinas x *L. serriola* acc. US96UC23 were a subset of the 213 RILs used to generate an ultra-dense, gene-based map using hybridization data from a custom Affymetrix GeneChip[2]. The same DNA samples were used to make libraries for sequencing the gene space of the RILs. Genomic DNA was isolated from leaves of the RILs using a modified CTAB extraction method[3]. DNA samples were fragmented to lengths of ~300 bp by sonication using an UCD-200 Bioruptor as per the manufacturer's instructions (Diagenode, Inc., Denville, NJ, USA). Genomic paired-end libraries were then prepared using standard procedures and NEB reagents for end-repair, A-tailing, and adapter ligation. All libraries were PCR amplified using the high-fidelity Phusion polymerase. Libraries were indexed using sequencing adapters with 6-bp in-line barcodes and pooled into three subsets of 33 RILs. Aliquots of the libraries were normalized using a Duplex-Specific Nuclease protocol[4]. The pooled libraries were sequenced to approximately 1x on an Illumina Hiseq 2000 sequencer.

Two Chicago libraries were prepared by Dovetail genomics as described previously[5]. Briefly, ≥ 0.5 µg of high molecular weight genomic DNA (~100 Kb mean fragment size) was extracted from seeds of *L. sativa* cv. Salinas, reconstituted into chromatin *in vitro*, and fixed with formaldehyde. Fixed chromatin was then digested with *Mbo*I, the 5' overhangs were filled in with biotinylated nucleotides, and then the free blunt ends were ligated. After ligation, the crosslinks were reversed and the DNA purified from protein. Purified DNA was treated to remove biotin that was not internal to ligated fragments. The DNA was sheared to ~350 bp mean fragment size and sequencing libraries were generated using NEBNext Ultra enzymes (New England Biolabs, Ipswich, Ma) and Illumina-compatible adapters. Biotin-containing fragments were then isolated using streptavidin beads before PCR enrichment of the library. Libraries were sequenced in two lanes on an Illumina HiSeq 2500 in rapid run mode to generate 313.5 M and 357.2 M 100 bp read pairs. This provided a total of 72x physical coverage.

Genome assemblies

The *L. sativa* genome was assembled using SOAPdenovo2[6] with parameter-k 43. The reads from the PE libraries were first assembled to obtain contigs, and then all reads from MP libraries were aligned onto the contigs to construct scaffolds. The amount of

shared paired-end relationships between each pair of contigs was calculated, the rate of consistent and conflicting paired-ends weighted, and then the scaffolds constructed reiteratively, in increasing order of insert size. These scaffolds contained gaps mainly due to repeat sequences that had been masked prior to the scaffold construction phase. To fill these gaps, paired-end information was used to retrieve the read pairs that had one end mapped to unique contigs and the other located in gaps and then local assemblies were performed using these reads to fill in the gaps.

The *L. sativa* draft genome in FASTA format (2,383 Mb with a scaffold N_{50} of 476 Kb), and the Chicago library sequences in FASTQ format were used as input data for HiRise, a software pipeline designed specifically for using Chicago library sequence data to assemble genomes[5]. Shotgun and Chicago library sequences were aligned to the draft input assembly using the SNAP read mapper (http://snap.cs.berkeley.edu). The separations of Chicago read pairs mapped within draft scaffolds were analyzed by HiRise to produce a likelihood model and the resulting likelihood model was used to identify putative misjoins and score prospective joins. After scaffolding, shotgun sequences were used to close gaps between contigs (HiRise scaffolding was preformed at Dovetail). Two iterations of HiRise scaffolding were done; the first used a single lane of sequencing from one Chicago library and the second used data from sequencing one lane of each two Chicago libraries. The quality of both assemblies were evaluated using in-house scripts.

Assembly validation and genetic analysis

Completeness of the assembly was evaluated using multiple datasets. The CEGMA pipeline[7] was used to asses the quality of the assembly with the conserved eukaryotic sequences; in parallel with a set of 357 Ultra Conserved Ortologous Sequences[8] (UCOS; available at http://compgenomics.ucdavis.edu/compositae_reference.php) from *A. thaliana* that was aligned with BlastX $1xe^{-20}$. EST sequences from NCBI for the *Lactuca* clade were also aligned to the genome using GMAP version 2014-12-17[9] by species. Presence/absence of an EST was determined after filtering the alignments by identity and coverage at different levels of stringency using custom scripts.

Reads were assigned to each of the 99 F_7 RILs from *L. sativa* cv. Salinas *x L. serriola* acc. US96UC23 based on their index sequences. Reads for each RIL were then mapped to the genomic sequence of *L. sativa* using CLC Genomics Server 6.5[10] (Qiagen, Redwood City, Ca, USA) and haplotypes assigned to each scaffold in each RIL based on the consensus all SNPs per scaffold. Low quality calls were filtered out using MadMapper[11] by summarizing scores across all RILs. Scaffolds that exhibited haplotypes with many discontinuities at the same position were considered to be chimeric erroneous assemblies (Suppl. Fig. 1). Reads were mapped back to these scaffolds to identify the position of mis-assembly and the scaffold split at that point. The two halves were subsequently analyzed independently. To map the scaffolds from *L. sativa*, all scaffolds over 1 Kb were clustered into nine chromosomal linkage groups (LGs) using MadMapper[11]. Within each chromosomal linkage group scaffolds were then assigned to genetic bins based on their segregation using MSTmap[12] (parameters: population_type = RIL7, distance_function = kosambi, cut_off_p_value = 1, no_mad_dist = 15, no_map_size = 2, detect_bad_data = yes,

objective_function = ML). For comparison of the new genetic map with the previous Affymetrix GeneChip map[2] unigenes used in the chip construction (available at http://chiplett.ucdavis.edu) were mapped to SoapDenovo scaffolds using GMAP version 2014-12-17[9]. Only unigenes with unique mappings were considered for correlation between the maps and filtered based in 60% identity and 60% coverage. Correlation was done by comparing the genetic position of the unigene in the ChipMap against the genetic location of the scaffold where the unigene maps in the new map.

Genetic information from the scaffold map was used for validation of the HiRise superscaffolds. For this purpose the genetic coordinates for each scaffold were aligned with the physical coordinates of the superscaffolds and used to classify the superscaffolds based in the relation between genetic and physical positions. Several characteristics were used to triage the data:

- Unmapped: Superscaffolds that did not contain any mapped scaffolds. These superscaffolds were not considered further.
- Single mapped scaffolds: Superscaffolds that contained only one mapped scaffold. These superscaffolds were not analyzed further.
- Single genetic bin: Superscaffolds where all the mapped scaffolds mapped to the same genetic location. These superscaffolds did not require further inspection.
- Chimeric: the chimeric scaffolds were divided into two categories. An outlier LG category consisted of superscaffolds with at least five mapped scaffolds that mapped to two LGs but one of the LG only had a single scaffold; these single scaffolds were just flagged as outliers. All other superscaffolds that mapped to two LG were flagged as chimeric. This latter set was inspected visually to identify the points of misjoin based in the location of the joint between the LGs.
- Validated: For superscaffolds that contained a single LG and span multiple genetic positions, were classified as validated if the genetic and physical positions agreed perfectly ($\pm$ 1 cM). These superscaffolds didn't required further inspection.
- Rearranged: Scaffolds that contained only 1 LG and spanned multiple cM but had discrepancies between the genetic and physical location (change in direction or large gaps (greater than 10 cM) between two adjacent scaffolds) were classified as rearranged scaffolds and were inspected visually to determine whether they needed to be split.

After validation and clean-up of the HiRise assembly, superscaffolds were mapped into the nine LG's. An initial order was assigned based on the genetic position of the first and last scaffolds within the superscaffold; validated superscaffolds were oriented based on their terminal locations. This order was revised based in the terminal haplotypes (SNP's within the last 10 Kb) of the superscaffolds from the 99 F_7 RILs from *L. sativa* cv. Salinas *x L. serriola* acc. US96UC23 to minimize the double recombinants and genetic inconsistencies by orienting superscaffolds in cases where that was possible and organizing the flanking superscaffolds of the genetic bins; the revised order of the superscaffolds was use to constructed chromosomal pseudomolecules joining superscaffolds with 10 Kb of N's as spacers. Telomeric sequence arrays were found by string searches of known telomere

sequences present in plants[13] across the pseudomolecules and determined based on the number of repeats found.

Prediction and analysis of repeat sequences

Transposable elements (TEs) were identified in the genome using a combination of homology-based and *de novo* approaches[6]. The genome was mined for repeat elements using ProteinMask and RepeatMasker[14] using Repbase[15] as reference. For identification of repeats *de novo*, a library of repeated sequences was built with RepeatModeler v.1.0.8[16] and RepeatScout[17]; similarly LTR_Finder[18] and MITE hunter[19] were used to generate libraries of long terminal repeat retrotransposons (LTR-RT) elements and miniature inverted-repeat transposable elements (MITEs), respectively. All custom libraries were then used to screen for more elements using RepeatMasker. Finally, TRF[20] was used to find tandem repeats. Repeat content along the genome was calculated using BedTools v2.17.0[21] coverage function with the predicteded repeat elements from all the sources.

Prediction of non-coding RNA genes

Prediction of ncRNA was done by type, first tRNAscan-SE[22] was use to predict tRNAs. Similarly snoscan[23] and RNAmmer[24] were used to predict snoRNA and rRNA respectively. Infernal[25] was use for prediction of more ncRNA using the rFam database[26] as input for miRNA, rRNA, rybozimes, snRNA and tRNA.

Small RNA and target gene analysis

Total RNA was isolated from leaves of *L. sativa* cvs. Salinas, Cobham Green, and Diana, infected or not infected by *Bremia lactucae* (10 samples total) using Concert™ Plant RNA Reagent (Invitrogen/Life Technologies, Carlsbad, CA). Small RNA libraries were constructed using the TruSeq Small RNA Sample Preparation Kit (Illumina, Hayward, CA). Parallel analysis of RNA end (PARE) libraries were constructed as previously described[27]. The libraries were sequenced on an Illumina HiSeq 2000 at the Delaware Biotechnology Institute (Newark, DE).

The raw reads of sRNA sequencing data were trimmed to remove adaptor sequences and then mapped to the lettuce genome using Bowtie[28]. Reads that matched tRNAs, rRNAs, snRNA, and snoRNAs were excluded. Only reads that perfectly matched the lettuce genome were used for further study.

MiRNA prediction was performed using the pipeline reported in Arikit[29]. Individual steps in the process were performed with Perl scripts combined with miREAP[30] and CentroidFold[31]. MiREAP was used to evaluate the pairing of the miRNA and miR* and CentroidFold was used with default settings to visualize the overall miRNA precursor structure for manual evaluation. The parameters for miREAP were set to allow a maximal distance of 400 nucleotides between miRNA and miRNA* (-d 400), extending 25 nucleotides at the end of the precursor (-f 25), with filters optimized for animal miRNAs turned off and including minor tuning for plant miRNA characteristics. The features to determine miRNAs include a single-strand bias ≥0.9 and an abundance bias ≥0.7 based on the features of conserved miRNAs.

PHAS analyses were conducted as indicated in Xia[32] and Arikit[29]. Briefly, a 189 bp sliding-window based approach was used to calculate the p-value of each region containing

small RNAs. Genomic loci with p-value <0.001 were selected and adjacent loci with a distance of <5 Kb were combined into a single locus. Sequence annotation of *PHAS* loci was conducted with BLASTX with e-value $<10^{-4}$.

Genome-wide miRNA target prediction was performed using the sPARTA package[33] with the built-in target-prediction module miRFerno with 'standard' scoring schema and score cutoff ≤7, followed by PARE-based validation of predicted targets. The threshold for the validated miRNA-target interactions was a corrected p-value <0.05 and with PARE read abundance ≥5 at the cleavage site.

Gene annotation

Protein-coding genes were predicted using multiple gene annotation pipelines. Gene models were initially generated with each pipeline and then combined to provide a final set of gene models. An initial set of gene models was generated by performing *ab initio* gene prediction using AUGUSTUS[34] and Glimmer HMM[35] on the repeat-masked genome, with the help of the HMM model. Homologous proteins of other species (*Arabidopsis thaliana*, *Teobroma cacao*, *Solanum tuberosum*, *Vitis vinifera*, *Glycine max*) were mapped to the genome using TblastN[36] with an E-value cutoff of 1 x e^{-5}. The aligned sequences, as well as their corresponding query proteins, were then filtered and passed to GeneWise[37] to search for accurately-spliced alignments. ESTs were aligned to the genome using BLAT[38] to generate spliced alignments. Then the spliced alignments were linked according to overlap using PASA[39]. The three lines of evidence generated from the above approaches were then integrated using GLEAN[40] to produce a consensus gene set. Transcripts were generated from RNAseq data using Tophat[41] and Cufflinks[42]. The GLEAN gene set was then analyzed to generate complete gene structures with UTRs using the transcriptome data. A second set of gene models was generated for *L. sativa* using the MAKER annotation pipeline[43]. Gene prediction datasets were collapsed based in the genomic coordinates of the gene models into loci. This was then reduced to a set of non-redundant gene models by eliminating multiple predictions with the same sequence, structure and location. An Overlap Weighted Evidence Gene-model (OWEG) score was calculated for all the gene models by performing a weighted sum of the amount of lines evidence that overlaps with each of the exons of the gene model (Reyes-Chin-Wo *et al*., unpublished). This score was use to select a gene model representing each locus and provide an indication of confidence for downstream analyses. The predicted proteome and genic region were evaluated with CEGMA[7] and against the UCOS[8] to evaluate their completeness. Annotation files for publicly available were downloaded from Phytozome V9[44] (*A. thaliana*, *T. cacao*, *Poplar trichocarpa*, *Solanum lycopersicum*, *G. max*), SolGenomics[45] (*Nicotiana tomentosis and Solanum pinnelli*) and from the Pepper Genome Database[46] (*Capsicum annum*). Gene statistics (transcript sizes, CDS sizes, exon sizes, intron sizes, number of exons per gene and intergenic distance) were calculated from the GFF files using custom scripts for all genomes. Tandem gene arrays were identified using the CoGe platform as one of the SynMap[47] outputs.

Protein clustering and functional annotation

Functional annotation of all the transcripts was done using InterProScan5[48] (with PANTHER v7.2, pFam v26.0, Prosite v20.89, PRINTS v42.0 and SMART v6.2) for annotation

of protein domains and GO annotations and KEGG Automated Annotation Service (KAAS)[49] for identification of KEGG enzymes; predicted proteins domains were filtered for a p-Value smaller than $1x10^{-3}$. Predicted proteins were BLASTed against *A. thaliana* TAIR10 proteins with blastp $1xe^{-10}$ to find homologous sequences.

The predicted proteome of *L. sativa* was clustered in OrthoMCL[50] following standard pipeline with proteins from *A. thaliana*, *Brassica rapa*, *Citrus sinensis*, *Fragaria vesca*, *Mimulus guttatus*, *P. trichocarpa*, *S. lycopersicum*, *T. cacao* and *V. vinifera*. Protein sequences for all plant species were downloaded from Phytozome V9[44]. Genes present in clusters that were single copy and present in all genomes were used to calculate divergence with *V. vinifera* as the reference. Pairwise comparisons were made by aligning the protein sequences for each species against *V. vinifera* ortologs using ClustalOmega v1.2.0[51]. The proteins alignments were converted to a codon alignment using Pal2Nal v14[52] and sequence divergence estimated with baseml and codeml from PAML v4.7a[53].

Chi-square tests were performed in selected gene clusters that were present in all species and had a mean number of genes/species greater than 5 to test whether gene copies were evenly distributed across the panel of ten species. Expected values were calculated multiplying the mean number of genes per family by a normalization factor (based on the total number of clustered genes per species). Tests were identified as significant with a 0.05 significance threshold with Bonferroni corrected p-values. Annotation from *L. sativa* was used to classify the clusters into protein functions. Receptor Like Kinases[54] (RLK) proteins were classified by having a protein kinase domain (PF00069, PF07714, PF12398, PS50011, SM00220, SM00219) and a transmembrane domain predicted by TMHMM v2.0 [55]. Genes that contain the kinase and the transmembrane domain were classified further based in the extracellular predicted domains [56] (LRR: PF00560, PF13504, PF13855, PF13516, PF08263, PF12799, PS51450, SM00365, SM00364, SM00369, L-Lectin: PF00139, B-Lectin: PS50927, SM00108).

Nucleotide binding-leucine rich repeat receptor like (NLR) information was overlaid on the clusters to identify resistance gene related groups using the gene ID's from Christopoulo[57]. This information was used to dissect the general patterns of expansion and contraction of the NLR gene sub-families, as well as presence of homologous genes in other plant species. Besides NLR-related groups the *L. sativa* annotation was used to define *LOX*-like genes using the pFam domain PF00305. For the selected group all protein sequences from the panel of species were aligned using ClustalOmega v1.2.0[51], this alignment was trimmed using JalView2[58] which was feed into Clustal Phylogeny for tree construction and visualized on the interactive Tree Of Life (iTOL)[59].

Clusters with only *L. sativa* genes were defined as specific to this species. All the genes within these clusters were selected and used for enrichment analysis with a fisher exact test on Blast2GO[60] using all predicted genes as reference set with an FDR threshold of 0.05. Resulting GO terms were filtered using the "Reduce to most specific" function and visualized with the GO network function.

In depth analysis of other protein families domains from Pfam database v27.0[61] were analyzed using hmmer version 3.1b1[62] to search predicted protein sequences download from Phytozome V9[44] for 9 genomes: *A. thaliana*, *B. rapa*, *C. sinensis*, *F. vesca*, *M. guttatus*, *P. trichocarpa*, *S. lycopersicum*, *T. cacao* and *V. vinifera*, and predicted protein sequences of *L. sativa*. Number of protein domains detected with hmmserach[62] with expectation value 1e^{-10} or better per species were compiled into a table and analyzed for an abundance of particular gene families in *L. sativa*.

Analysis of synteny

Synteny analyses were done in the CoGe platform (https://genomeevolution.org/) using SynMap[47]. Parameters were optimized to work with rearranged and conserved genomes using *A. thaliana* and *P. trichocarpa* as benchmarks. Analysis options that were modified were "Merge Syntenic Blocks" with the "Quota Align Merge" algorithm; "Maximun distance between two blocks" was set to 2 and C-score filter was set to 0.3, remaining options were left as default.

Comparisons were done using *V. vinifera* and *L. sativa* as references against 15 publicly available plant genomes on CoGe (provide the CoGe genome ID as reference) (*Actinidia chinensis* (ID20457), *Aquilegia coerulea* (ID10706), *A. thaliana* (ID18626), *B. rapa* (ID22733), *C. annum* (ID28029), *Coffea canephora* (19433), *C. sinensis* (ID19805), *F. vesca* (ID14154), *G. max* (ID14154), *M. guttatus* (ID8156), *P. trichocarpa* (ID19384), *S. lycopersicum* (ID27980), *S. tuberosum* (ID27982), *T. cacao* (ID10997) and *Utricularia gibba* (ID19456)), synteny was also calculated to *Cynara cardunculus*[63] as another Compositae species. Besides synteny against *V. vinifera and L. sativa* intragenomic synteny was calculated for all Asterid genomes. SynMap results were parsed with in house scripts to extract syntelog information and calculate syntenic statistics (total numbers of syntenic genes, total number of blocks, mean block size, variance in block size. For all the comparisons against lettuce and for the intragenomic comparisons sequence divergence estimates for syntelogs pairs was calculated using the same protocol as used for the OrthoMCL ortologs.

A phylogenetic tree was constructed using sequences of predicted CEG genes using CEGMA[7] on all the Asterids species plus *A. thaliana*, *M. truncatula, P. trychocarpa, T. cacao, E. grandis, V. vinifera* and *A.coerulea* using the sequences downloaded from Phytozome v9. All sequences that were predicted as complete in all species were aligned using Clustal Omega v1.2.0[51] by CEG. These alignments were inspected visually using JalView[58] to remove problematic areas. After clean up all alignments were concatenated and fed into Mega v5.1[64] to determine the best substitution model. Concatenated alignment was then use to construct a maximum likelihood tree in RAxML v8.0.26[65] using the selected model (parameters -p 12345 -x 12345 -N 100 -m PROTGAMMAIJTT -f a), and then visualized using TreeGraph 2.1.0[66].

Triplicated blocks in the *L. sativa* genome were identified by aligning syntenic blocks to the reference and extracting regions that had at least five syntenic genes across two blocks. These putative triplicated blocks were visually inspected and collapsed into

regions by studying their pattern along the genome; to collapse two blocks into a triplicated region they had to be less than 50 genes apart and shared the same chromosome set. Using the syntelogs on the triplicated blocks the estimated time for the duplication events was estimated using the formula T = d/r, using the estimated sequence divergence by baseml from the PAML package and r = 1.0×10^{-8} as previously calculated for host-encoded genes[67]. Synonymous substitution rates were used to study the divergence pattern between the paralogous regions and also group them into the three subgenomes.

All blocks for each triplicated region were extracted and combined based in their genomic location, blocks that belonged to the same region where joined if they were less than 20 Kb apart. These combined blocks were then used for calculation of triplication statistics, gene content and repeat content were calculated using the annotation files and BedTools v2.17.0[21] coverage function. The combined blocks were also used to determine genes within the triplicated regions using BedTools intersect function. GO enrichment analysis was performed on Blast2GO with a fisher exact test using the genes within triplicated regions against the whole genome to test for differences in gene type distribution with an FDR of 0.05. Results were summarized using the "Reduce to most specific" function and visualized with GO network function. For all genes within triplications a count of syntenic mates was generated to identify the degree of conservation (no extra copies, retain of one copy or retain of the two extra copies). Using this information a second GO enrichment on Blast2Go was done using the genes that have retain at least one copy against all the genes within the triplicated regions.

For further analysis 4 transcription families were selected based in their biological significance (WRKY (PF03106), AP2 (PF00847), TCP (PF03634), MADS (PF00319)). All the genes were extracted from each of these families from the entire genome and syntelogs were designated when both contained the same domain(s). For these syntelogs, proteins were split into N and C termini at the start of the first transcription factor domain. Mutation rates were calculated for both termini per syntelog pair using the same approach as for the entire syntelog. In parallel, all the protein sequences for each family were aligned with Clustal Omega v1.2.0[51] and the alignment imported into web version of Clustal Phylogeny[68] for tree construction using default parameters. The tree was visualized and annotated in iTOL with degree of gene retention. *TCP-tf* gene family was further investigated for identification of the *Cycloidea*-like genes[69]. All the *Cycloidea*-like sequences for the Asteraceae clade with "compelte cds" were download from NCBI, aligned to the predicted *TCP-tf* using blastn $1x^{-10}$ and alignment results cleaned visually. These hits were used to identify the *Cycloidea*-like clade in the *TCP-tf* gene tree.

Expression data for the genes within the triplicated regions was gathered for 137 samples spread across three experiments to study the expression patterns of syntelogs[70–72]. The RNA-seq reads were mapped against the *L. sativa* superscaffolds using TopHat[73]. Read counts for the predicted genes was calculated with BedTools multicov function for all samples. All data was imported into the R v3.2.3 environment[74]. Due to the high level of variation read counts were normalized, for this purpose size factors were calculated with the DeSEQ2 package[75] "estimateSizeFactors" function and then applied to the different

samples. Filtering was done after normalization for genes with more than 10 counts on at least 25% of the samples. Clustering of filtered normalized expression values was done with the Weighted Gene Co-Expression Network Analysis package[76], following the tutorials for step-by-step network construction (network construction parameters: power = 8, minclustersize = 30, deepsplit = 3, cutdist = 0.3). After network construction syntelogs were determined as co-expressed if they belong to the same module.

Supplementary References

1. Doyle, J. & Doyle, J. L. Genomic plant DNA preparation from fresh tissue-CTAB method. *Phytochem Bull* **19,** 11–15 (1987).

2. Truco, M. J. *et al.* An ultra high-density, transcript-based, genetic map of Lettuce. *G3 Genes|Genomes|Genetics* **3,** 617–631 (2013).

3. Rogers, S. O. & Bendich, A. J. Extraction of DNA from plant tissues. *Plant Mol. Biol. Man.* 73–83 (1988). doi:10.1007/978-94-009-0951-9_6

4. Matvienko, M. *et al.* Consequences of normalizing transcriptomic and genomic libraries of plant genomes using a duplex-specific nuclease and tetramethylammonium chloride. *PLoS One* **8,** e55913 (2013).

5. Putnam, N. H. *et al.* Chromosome-scale shotgun assembly using an *in vitro* method for long-range linkage. *Genome Res.* (2016). doi:10.1101/gr.193474.115

6. Li, R. *et al. De novo* assembly of human genomes with massively parallel short read sequencing. *Genome Res.* **20,** 265–272 (2010).

7. Parra, G., Bradnam, K. & Korf, I. CEGMA: A pipeline to accurately annotate core genes in eukaryotic genomes. *Bioinformatics* **23,** 1061–1067 (2007).

8. Kozik, A. *et al.* Eukaryotic ultra conserved orthologs and estimation of gene capture In EST libraries. in *Plant and Animal Genomes Conference* **16,** P6 (2008).

9. Wu, T. D. & Watanabe, C. K. GMAP: a genomic mapping and alignment program for mRNA and EST sequences. *Bioinformatics* **21,** 1859–1875 (2005).

10. CLC Bio. CLC Genomics Server.

11. Kozik, A. Suite of python MadMapper scripts for quality control of genetic markers, group analysis and inference of linear order of markers on linkage groups. (2006). Available at: http://cgpdb.ucdavis.edu/XLinkage/MadMapper/.

12. Wu, Y., Bhat, P. R., Close, T. J. & Lonardi, S. Efficient and accurate construction of genetic linkage maps from the minimum spanning tree of a graph. *PLoS Genet* **4,** e1000212 (2008).

13. Podlevsky, J. D., Bley, C. J., Omana, R. V, Qi, X. & Chen, J. J.-L. The Telomerase Database. *Nucleic Acids Res.* **36,** D339–D343 (2008).

14. Smit, A., Hubley, R. & Green, P. RepeatMasker Open-3.0. *RepeatMasker Open-3.0*

15. Jurka, J. *et al.* Repbase Update, a database of eukaryotic repetitive elements. *Cytogenet. Genome Res.* (2005).

16. Smit, A. & Hubley, R. Repeat Modeler - 1.0.8. Available at: http://www.repeatmasker.org/RepeatModeler.html.

17. Price, A. L., Jones, N. C. & Pevzner, P. A. *De novo* identification of repeat families in

large genomes. *Bioinformatics* **21,** i351–i358 (2005).

18. Xu, Z. & Wang, H. LTR_FINDER: an efficient tool for the prediction of full-length LTR retrotransposons. *Nucleic Acids Res.* (2007). doi:10.1093/nar/gkm286

19. Han, Y. & Wessler, S. R. MITE-Hunter: A program for discovering miniature inverted-repeat transposable elements from genomic sequences. *Nucleic Acids Res.* **38,** e199 (2010).

20. Benson, G. Tandem repeats finder: a program to analyze DNA sequences. *Nucleic Acids Res.* (1999).

21. Quinlan, A. R. & Hall, I. M. BEDTools: A flexible suite of utilities for comparing genomic features. *Bioinformatics* **26,** 841–842 (2010).

22. Lowe, T. M. & Eddy, S. R. tRNAscan-SE: A Program for Improved Detection of Transfer RNA Genes in Genomic Sequence. *Nucleic Acids Res.* (1997). doi:10.1093/nar/25.5.0955

23. Lowe, T. M. & Eddy, S. R. A Computational Screen for Methylation Guide snoRNAs in Yeast. *Sci.* (1999). doi:10.1126/science.283.5405.1168

24. Lagesen, K. *et al.* RNAmmer: consistent and rapid annotation of ribosomal RNA genes. *Nucleic Acids Res.* (2007). doi:10.1093/nar/gkm160

25. Nawrocki, E. P. & Eddy, S. R. Infernal 1.1: 100-fold faster RNA homology searches. *Bioinforma.* **29,** 2933–2935 (2013).

26. Nawrocki, E. P. *et al.* Rfam 12.0: Updates to the RNA families database. *Nucleic Acids Res.* **43,** D130–D137 (2015).

27. Zhai, J. *et al.* MicroRNAs as master regulators of the plant NB-LRR defense gene family via the production of phased, trans-acting siRNAs. *Genes Dev.* **25,** 2540–2553 (2011).

28. Langmead, B., Trapnell, C., Pop, M. & Salzberg, S. L. Ultrafast and memory-efficient alignment of short DNA sequences to the human genome. *Genome Biol.* **10,** 1 (2009).

29. Arikit, S. *et al.* An atlas of soybean small RNAs identifies phased siRNAs from hundreds of coding genes. *Plant Cell* **26,** 4584–4601 (2014).

30. Qibin, L. & Jiang, W. MIREAP: microRNA discovery by deep sequencing. (2013). Available at: https://sourceforge.net/projects/mireap/.

31. Sato, K., Hamada, M., Asai, K. & Mituyama, T. CENTROIDFOLD: a web server for RNA secondary structure prediction. *Nucleic Acids Res.* **37,** W277–W280 (2009).

32. Xia, R. *et al.* MicroRNA superfamilies descended from miR390 and their roles in secondary small interfering RNA biogenesis in Eudicots. *Plant Cell* **25,** 1555–1572 (2013).

33. Kakrana, A., Hammond, R., Patel, P., Nakano, M. & Meyers, B. C. sPARTA: a parallelized pipeline for integrated analysis of plant miRNA and cleaved mRNA data

sets, including new miRNA target-identification software. *Nucleic Acids Res.* (2014). doi:10.1093/nar/gku693

34. Stanke, M. *et al.* AUGUSTUS: *Ab initio* prediction of alternative transcripts. *Nucleic Acids Res.* **34,** W435-9 (2006).

35. Majoros, W. H., Pertea, M. & Salzberg, S. L. TigrScan and GlimmerHMM: Two open source *ab initio* eukaryotic gene-finders. *Bioinformatics* **20,** 2878–2879

36. Altschul, S. F., Gish, W., Miller, W., Myers, E. W. & Lipman, D. J. Basic local alignment search tool. *J. Mol. Biol.* (1990). doi:http://dx.doi.org/10.1016/S0022-2836(05)80360-2

37. Birney, E., Clamp, M. & Durbin, R. GeneWise and Genomewise. *Genome Res.* (2004). doi:10.1101/gr.1865504

38. Kent, W. J. BLAT—The BLAST-Like Alignment Tool. *Genome Res.* (2002). doi:10.1101/gr.229202

39. Haas, B. J. *et al.* Improving the *Arabidopsis* genome annotation using maximal transcript alignment assemblies. *Nucleic Acids Res.* **31,** 5654–5666 (2003).

40. Mackey, A., Liu, Q., Pereira, F. & Roos, D. GLEAN-Improved eukaryotic gene prediction by statistical consensus of gene evidence. *Genome Informatics* (2005).

41. Trapnell, C. *et al.* Differential gene and transcript expression analysis of RNA-seq experiments with TopHat and Cufflinks. *Nat. Protoc.* **7,** 562–78 (2012).

42. Trapnell, C. *et al.* Transcript assembly and quantification by RNA-Seq reveals unannotated transcripts and isoform switching during cell differentiation. *Nat. Biotechnol.* **28,** 511–515 (2010).

43. Holt, C. & Yandell, M. MAKER2: an annotation pipeline and genome-database management tool for second-generation genome projects. *BMC Bioinformatics* (2011). doi:10.1186/1471-2105-12-491

44. Nordberg, H. *et al.* The genome portal of the Department of Energy Joint Genome Institute: 2014 updates. *Nucleic Acids Res.* **42,** D26–D31 (2014).

45. Fernandez-Pozo, N. *et al.* The Sol Genomics Network (SGN)—from genotype to phenotype to breeding. *Nucleic Acids Res.* (2014). doi:10.1093/nar/gku1195

46. The pepper genome database (release 2.0). (2014).

47. Lyons, E., Pedersen, B., Kane, J. & Freeling, M. The value of nonmodel genomes and an example using SynMap within CoGe to dissect the hexaploidy that predates the Rosids. *Trop. Plant Biol.* **1,** 181–190 (2008).

48. Jones, P. *et al.* InterProScan 5: Genome-scale protein function classification. *Bioinformatics* (2014). doi:10.1093/bioinformatics/btu031

49. Moriya, Y., Itoh, M., Okuda, S., Yoshizawa, A. C. & Kanehisa, M. KAAS: an automatic genome annotation and pathway reconstruction server. *Nucleic Acids Res.* **35,**

W182–W185 (2007).

50. Fischer, S. *et al.* Using OrthoMCL to assign proteins to OrthoMCL-DB groups or to cluster proteomes into new ortholog groups. *Curr. Protoc. Bioinforma.* (2002). doi:10.1002/0471250953.bi0612s35

51. Dineen, S. A. *et al.* Fast, scalable generation of high-quality protein multiple sequence alignments using Clustal Omega. *Mol. Syst. Biol.* **7,** (2011).

52. Suyama, M., Torrents, D. & Bork, P. PAL2NAL: Robust conversion of protein sequence alignments into the corresponding codon alignments. *Nucleic Acids Res.* **34,** W609–W612 (2006).

53. Yang, Z. PAML 4: Phylogenetic analysis by maximum likelihood. *Mol. Biol. Evol.* **24,** 1586–1591 (2007).

54. Walker, J. C. Structure and function of the receptor-like protein kinases of higher plants. *Plant Mol. Biol.* **26,** 1599–1609 (1994).

55. Krogh, A., Larsson, B., Von Heijne, G. & Sonnhammer, E. L. L. Predicting transmembrane protein topology with a hidden Markov model: Application to complete genomes. *J. Mol. Biol.* **305,** 567–580 (2001).

56. Shiu, S.-H. & Bleecker, A. B. Plant receptor-like kinase gene family: Diversity, function, and signaling. *Sci. Signal.* **2001,** re22-re22 (2001).

57. Christopoulou, M. *et al.* Genome-wide architecture of disease resistance genes in Lettuce. *G3 Genes|Genomes|Genetics* (2015). doi:10.1534/g3.115.020818

58. Waterhouse, A. M., Procter, J. B., Martin, D. M. A., Clamp, M. & Barton, G. J. Jalview Version 2—A multiple sequence alignment editor and analysis workbench. *Bioinformatics* **25,** 1189–1191 (2009).

59. Letunic, I. & Bork, P. Interactive Tree Of Life v2: Online annotation and display of phylogenetic trees made easy. *Nucleic Acids Res.* **39,** W475–W478 (2011).

60. Conesa, A. *et al.* Blast2GO: A universal tool for annotation, visualization and analysis in functional genomics research. *Bioinformatics* **21,** 3674–3676 (2005).

61. Finn, R. D. *et al.* Pfam: The protein families database. *Nucleic Acids Res.* **42,** D222–D230 (2014).

62. Finn, R. D., Clements, J. & Eddy, S. R. HMMER web server: interactive sequence similarity searching. *Nucleic Acids Res.* **39,** W29–W37 (2011).

63. Scaglione, D. *et al.* The genome sequence of the outbreeding globe artichoke constructed de novo incorporating a phase-aware low-pass sequencing strategy of F1 progeny. *Sci. Rep.* **6,** 19427 (2016).

64. Kumar, S., Tamura, K., Jakobsen, I. B. & Nei, M. MEGA2: molecular evolutionary genetics analysis software. *Bioinforma.* **17,** 1244–1245 (2001).

65. Stamatakis, A., Ludwig, T. & Meier, H. RAxML-III: a fast program for maximum

likelihood-based inference of large phylogenetic trees. *Bioinforma.* **21,** 456–463 (2005).

66. Stöver, B. C. & Müller, K. F. TreeGraph 2: Combining and visualizing evidence from different phylogenetic analyses. *BMC Bioinformatics* **11,** 7 (2010).

67. Strasburg, J. L. & Rieseberg, L. H. Molecular demographic history of the annual sunflower *Helianthus annus and H. petiolaris*— Large effective population sizes and rates of long-term gene flow. *Evolution (N. Y).* **62,** 1936–1950 (2008).

68. McWilliam, H. *et al.* Analysis tool web services from the EMBL-EBI. *Nucleic Acids Res.* **41,** W597–W600 (2013).

69. Chapman, M. A., Leebens-Mack, J. H. & Burke, J. M. Positive selection and expression divergence following gene duplication in the sunflower *Cycloidea* gene family. *Mol. Biol. Evol.* **25,** 1260–1273 (2008).

70. De Cremer, K. *et al.* RNAseq-based transcriptome analysis of *Lactuca sativa* infected by the fungal necrotroph *Botrytis cinerea*. *Plant. Cell Environ.* **36,** 1992–2007 (2013).

71. Yoong, F.-Y. *et al.* Genetic variation for thermotolerance in lettuce seed germination Is associated with temperature-sensitive regulation of Ethylene Response Factor1 (*ERF1*). *Plant Physiol.* **170,** 472–488 (2016).

72. Macias González, M. Marker-trait association study in new RIL population from a cross between two Iceberg Lettuce cultivar. in *Plant and Animal Genome XXIV Conference* (Plant and Animal Genome).

73. Trapnell, C., Pachter, L. & Salzberg, S. L. TopHat: discovering splice junctions with RNA-Seq. *Bioinformatics* **25,** 1105–1111 (2009).

74. R Core Team. R: A Language and Environment for Statistical Computing. Available at: https://www.r-project.org.

75. Love, M. I., Huber, W. & Anders, S. Moderated estimation of fold change and dispersion for RNA-seq data with DESeq2. *Genome Biol.* **15,** 1–21 (2014).

76. Langfelder, P. & Horvath, S. WGCNA: an R package for weighted correlation network analysis. *BMC Bioinformatics* **9,** 1 (2008).
